# ‘Clip with Line Pulley Securing’ Technique using Modified Anchoring Clip for Mucosal Defect Closure

**DOI:** 10.1055/a-2134-6626

**Published:** 2023-08-21

**Authors:** Darshan Parekh, Yohei Minato, Nao Takeuchi, Shunya Takayanagi, Marina Kim, Suryaprakash Bhandari, Ken Ohata

**Affiliations:** 1 Department of Endoscopy, NTT Medical Center Tokyo, Tokyo, Japan; 2 Department of Endoscopy, Thane Institute of Gastroenterology, Thane, Maharashtra, India; 3 Division of Gastroenterology & Hepatology, UMass Memorial Medical Center, Worcester, Massachusetts, USA

Mucosal closure techniques are the backbone of interventional endoscopy, requiring focused and dedicated development. We present a novel closure technique that is easily applicable, economical, and effective for large mucosal defects.

An 85-year-man underwent gastric endoscopic submucosal dissection for adenocarcinoma of the stomach, which resulted in an ulcer measuring around 35 × 25 mm in size ( Fig. 1 ), and this was closed using the novel “CLiPS technique” ( Video 1 ).

**Fig. 1:**
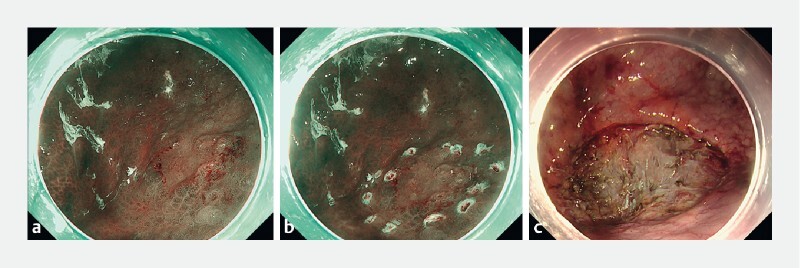
Endoscopic images during endoscopic submucosal dissection (ESD) showing: **a** the tumor; **b** marking around the tumor; **c** the ulcer following ESD (approximately 35 × 25 mm).

**Video 1** Endoscopic closure of a postendoscopic submucosal dissection ulcer using the novel “CLiPS technique.”


A modified anchoring clip was created by cutting the jaws off a large caliber reopenable clip (opening width 16 mm) and smoothing the edges with a file ( Fig. 2 a, b ). A clip line ( Fig. 2 c ) was prepared by tying a nylon thread (0.23-mm diameter) between the teeth of another reopenable clip and passing it through the accessory channel of a standard endoscope. The clip line was fixed at the distal edge, base, and proximal edge of the ulcer ( Fig. 3 a, b ). The line was then passed externally through the gap at the base of the modified clip, which was then inserted and was used to anchor the clip that had been placed on the proximal edge of the ulcer. A pulley system was thereby created that allowed the edges to be securely approximated ( Fig. 3 c, d ) using external countertraction on the line. The clip was deployed making the ulcer more linear and amenable to closure with standard hemoclips ( Fig. 4 ). The line was cut close to the anchoring clip and the defect was then closed completely with hemoclips ( Fig. 5 ).

**Fig. 2:**
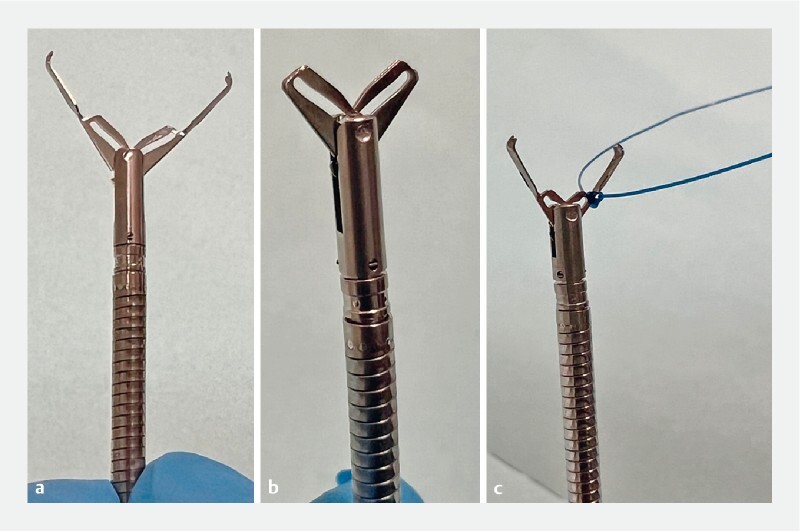
Photographs of: **a** the unmodified anchoring clip (opening width 16 mm); **b** the modified clip; **c** the clip line.

**Fig. 3:**
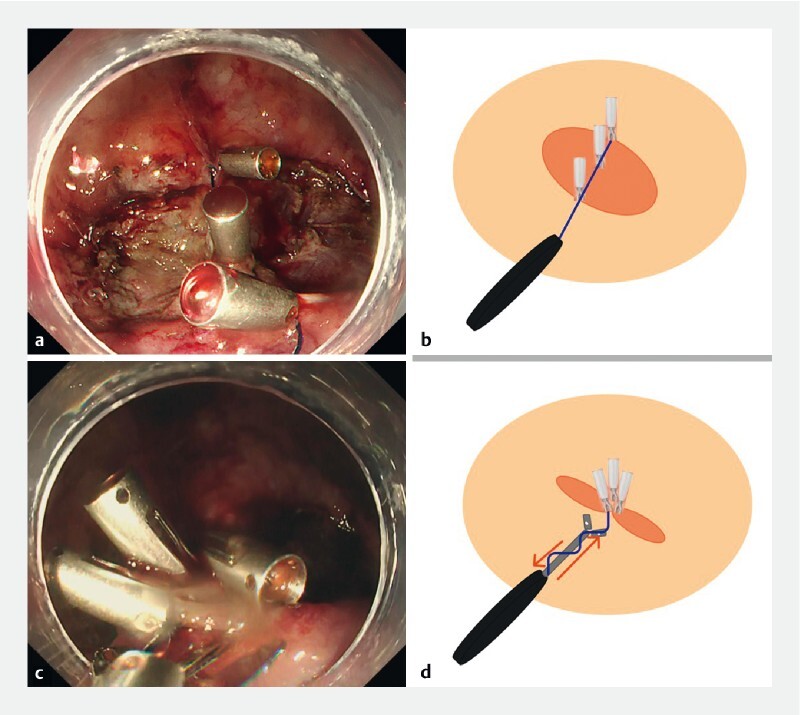
Images of the novel CliPs technique with: **a, b** the line fixed securely to the ulcer base and both edges; **c, d** the pulley system that allows the edges to be approximated by applying external countertraction to the line, as shown on: **a, c** endoscopic view; **b, d** schematics.

**Fig. 4:**
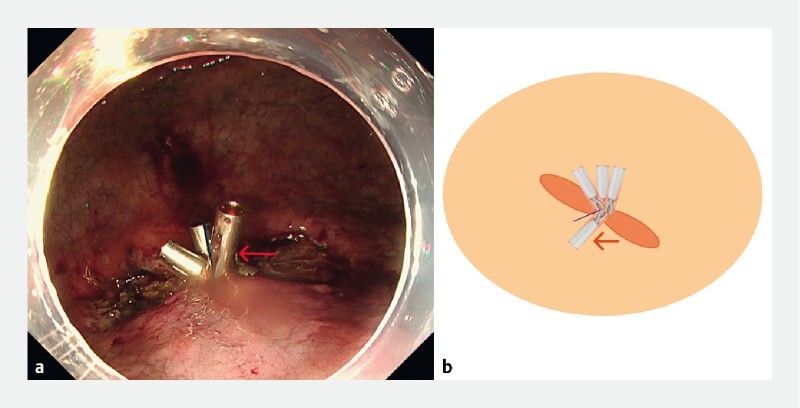
Deployment of the anchoring clip (arrow) making the ulcer amenable to closure with standard hemoclips is shown on: **a** endoscopic view; **b** a schematic.

**Fig. 5:**
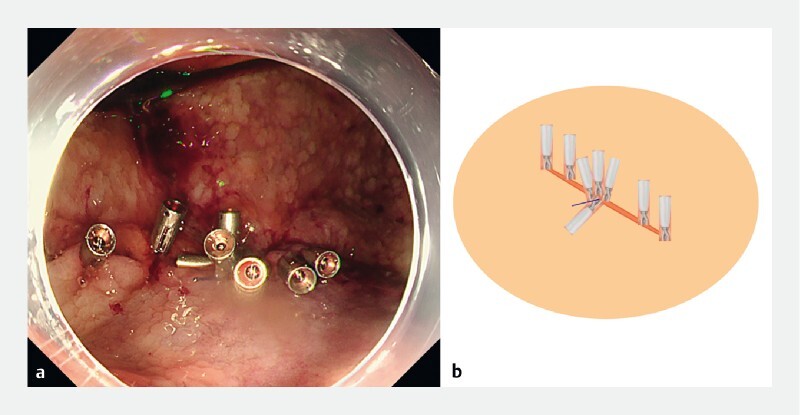
Complete closure of the defect is shown on: **a** endoscopic view; **b** a schematic.

The principle of the “CLiPS technique” is reduction in the defect size by strong and stable approximation of the edges using the pulley system. The anchoring clip supports the line and makes it independent of the scope, thereby increasing maneuverability of the scope. Our technique requires no additional accessories or special endoscopes. It does not require scope reinsertion and enables free maneuverability; it may be used in full-thickness closure. Further studies with more patients and larger defects should be considered.

Endoscopy_UCTN_Code_TTT_1AO_2AG

## Notes

Endoscopy E-Videos

https://eref.thieme.de/e-videos
*E-Videos* is an open access online section of the journal *Endoscopy* , reporting on interesting cases and new techniques in gastroenterological endoscopy. All papers include a high-quality video and are published with a Creative Commons CC-BY license. Endoscopy E-Videos qualify for HINARI discounts and waivers and eligibility is automatically checked during the submission process. We grant 100% waivers to articles whose corresponding authors are based in Group A countries and 50% waivers to those who are based in Group B countries as classified by Research4Life (see: https://www.research4life.org/access/eligibility/ ). This section has its own submission website at https://mc.manuscriptcentral.com/e-videos

